# Identification of dysfunctional modules and disease genes in congenital heart disease by a network-based approach

**DOI:** 10.1186/1471-2164-12-592

**Published:** 2011-12-02

**Authors:** Danning He, Zhi-Ping Liu, Luonan Chen

**Affiliations:** 1 Key Laboratory of Systems Biology, SIBS-Novo Nordisk Translational Research Centre for PreDiabetes, Shanghai Institutes for Biological Sciences, Chinese Academy of Sciences, Shanghai 200031, China; 2 Department of Health Sciences Informatics, Johns Hopkins University School of Medicine, Baltimore, MD 21205, USA; 3 Collaborative Research Center for Innovative Mathematical Modelling, Institute of Industrial Science, University of Tokyo, Tokyo 153-8505, Japan

## Abstract

**Background:**

The incidence of congenital heart disease (CHD) is continuously increasing among infants born alive nowadays, making it one of the leading causes of infant morbidity worldwide. Various studies suggest that both genetic and environmental factors lead to CHD, and therefore identifying its candidate genes and disease-markers has been one of the central topics in CHD research. By using the high-throughput genomic data of CHD which are available recently, network-based methods provide powerful alternatives of systematic analysis of complex diseases and identification of dysfunctional modules and candidate disease genes.

**Results:**

In this paper, by modeling the information flow from source disease genes to targets of differentially expressed genes via a context-specific protein-protein interaction network, we extracted dysfunctional modules which were then validated by various types of measurements and independent datasets. Network topology analysis of these modules revealed major and auxiliary pathways and cellular processes in CHD, demonstrating the biological usefulness of the identified modules. We also prioritized a list of candidate CHD genes from these modules using a guilt-by-association approach, which are well supported by various kinds of literature and experimental evidence.

**Conclusions:**

We provided a network-based analysis to detect dysfunctional modules and disease genes of CHD by modeling the information transmission from source disease genes to targets of differentially expressed genes. Our method resulted in 12 modules from the constructed CHD subnetwork. We further identified and prioritized candidate disease genes of CHD from these dysfunctional modules. In conclusion, module analysis not only revealed several important findings with regard to the underlying molecular mechanisms of CHD, but also suggested the distinct network properties of causal disease genes which lead to identification of candidate CHD genes.

## Background

Congenital heart disease (CHD) is among the most common human congenital defects, and is the leading cause of infant morbidity in the world [1,2]. Although CHD is known to arise from abnormal heart development during embryogenesis [3,4], its molecular mechanism remains far from clear. Currently, about 30 different genes have been known to cause CHD. Understanding the molecular functions, molecular interactions and represented pathways implicated in these CHD genes contribute to our knowledge of CHD pathogenesis, and therefore help improve clinical diagnosis and medical care of this disease. Network-based methods are powerful tools of systematic analysis of complex diseases, and identification of major pathways, responsive modules and candidate genes [5]. Previous works used those approaches to analyze heart development and cardiovascular disorders [6,7], however, there is no such study on CHD due to lack of its genome-wide expression data, which is publicly available only until very recently [8,9]. Therefore, in this paper by exploring high-throughput genomic data to elucidate essential roles of local network structures in CHD progression, we aim to provide such a network-based study on CHD as well.

To discover molecular pathogenesis in complex disease, considerable efforts have been made to elucidate the relations between variability in gene expression and genotype [10-12], and putative disease genes curated from literature research can be regarded as the source of molecular perturbations while differentially expressed genes identified from mRNA profiling can represent the responsive components of source perturbations. It is also noted that disease genes are not necessarily differentially expressed [13]. Differential gene expression level represents the changed phenotype that is potentially associated with the causal disease genes. Hence, linking causal disease genes with responsive differentially expressed genes by modeling the information flow in protein interactome can better reveal dysfunctional subnetworks and help the identification of disease modules. Previous research has established the analogy between random walks and electric networks [10,14]. Doyle and Snell [14] showed that when a unit current flow enters a source node and leaves a sink in the circuit network, the amount of current passing through any intermediary node or edge is proportional to the expected number of times the random walker visits that node or edge. Then, the amount of current passing though each node can be computed by solving a system of linear equations based on Kirchhoff's and Ohm's laws. Several recent studies also used such circuit flow networks to discover causal genes and associated pathways or to analyze gene network centrality [10,11,15,16].

In this paper, we construct a CHD subnetwork and identify dysfunctional modules by developing a novel network-based computational approach which integrates protein-protein interactions, gene coexpression profiles and causal paths from putative CHD genes to target genes. We evaluate the functional implications of our modules for phenotype classification, and further reveal their higher order topological relationships by exploring their represented biological processes and crosstalk. Results show that our modules are better disease-markers than documented pathways, and have the discriminative power stably across several independent microarray datasets. In particular, correlation analysis reveals that each module is also a group of significantly coexpressed genes; module interaction analysis characterizes the higher-order topology of these identified modules; functional enrichment and module-pathway crosstalk analysis suggests that the topology of a module is highly related to its roles in CHD. While the modules in central place of the CHD subnetwork are enriched in core CHD-related dysfunctional processes, such as anatomical structure morphogenesis, cell differentiation and cytoskeleton organization, and regulate key pathways of CHD such as cardiac muscle contraction, Notch signaling pathway and ECM (extracellular matrix)-receptor interaction, the modules in peripheral place are enriched in auxiliary processes, such as cell communication and various metabolic processes, and regulate less disease-related pathways. In addition, we find that CHD casual genes exhibit different network features, i.e. disease genes tend to have lower current flow and participate in fewer dysfunctional subnetworks than expected. Moreover, we provide a list of candidate CHD genes by module analysis, where the top ranked genes in the candidate list are all well supported by literature and experiment evidence. The results not only elucidate the functional roles of the modules on CHD, but also provide some insights into the underlying molecular mechanisms of CHD which lead to identification of candidate CHD genes.

## Results

### Identified dysfunctional modules

**Figure 1:**
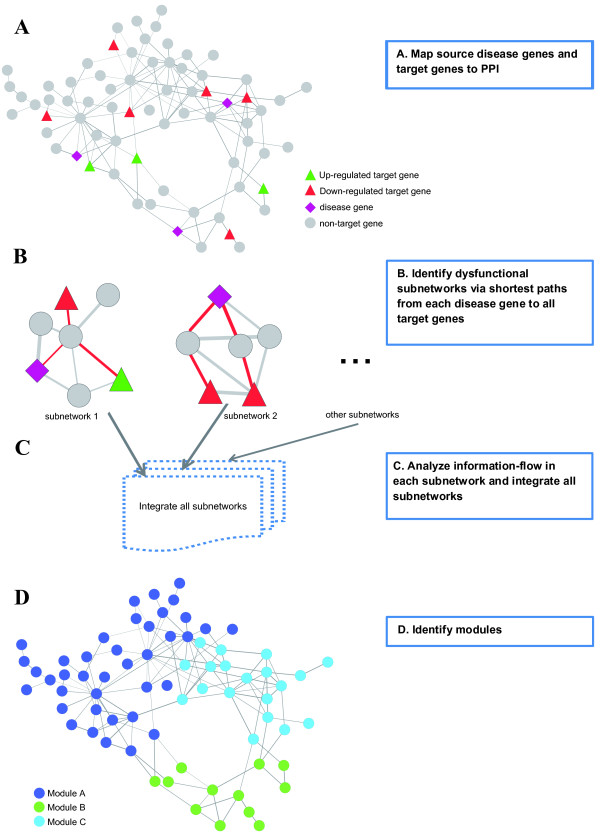
**Method Overview**. Triangles represent disease genes, rectangles represent target genes and grey circles represent other genes on the PPI network. (A) We overlaid expression profiles to PPI network, and mapped putative disease genes and differentially expressed target genes onto this weighted PPI network. (B) For each disease gene, we found its shortest paths to all target genes. (C) We computed the information flow for each node in a given subnetwork, and genes participating in several subnetworks can have several information scores. (D) We identified modules by assigning a gene to the module in which its information score is maximum.

The work flow of our method of identifying dysfunctional modules is shown in Figure 1. To capture the information flow from causal genes to target genes and to identify dysfunctional modules from these causal paths, we first identified 85 target genes which are defined as those differentially expressed (DE) in sufficient proportion of patients (Additional File 1), and then connected each known causal genes of CHD with these target DE genes via shortest paths shown in Figure 1A and 1B. We model the protein interaction network as an electrical circuit where correlation coexpression of two end nodes of an interaction is used as the conductance of a resistor, and biological signals from disease genes propagate through PPI edges to responsive genes just like electrical current flows through resistors [11,15]. Information from source genes will propagate its effect via protein-protein interactions, and DE genes which cover the majority of patients represent common dysregulated pathways in CHD. In the third step, we merged the paths from one causal gene to all target genes into a subnetwork, and computed the current flow for each gene to evaluate its importance in this local subnetwork as shown in Figure 1C. To measure the importance of one node in conducting electrical current, we computed the current flows through the node using the electronic laws [14], and defined the information flow score of the node as the sum of current through the node among all pair-wise combinations of the source node (the causal disease) and all target nodes (all responsive differentially expressed genes). Since the causal subnetwork can overlap, i.e. a gene can have several current flow scores, we assigned the gene to the subnetwork in which its current flow is maximum to derive mutually exclusive modules. The modules can also be thought of as an information-processing unit [5,17], therefore we put the gene into the module where the largest amount of information passes through it. At last, reasoning that highly connected genes are more likely to coordinate and perform the same biological functions, we pruned the modules and iterated this process until each of them was connected as shown is Figure 1D (further details can be referred to Methods).

**Figure 2:**
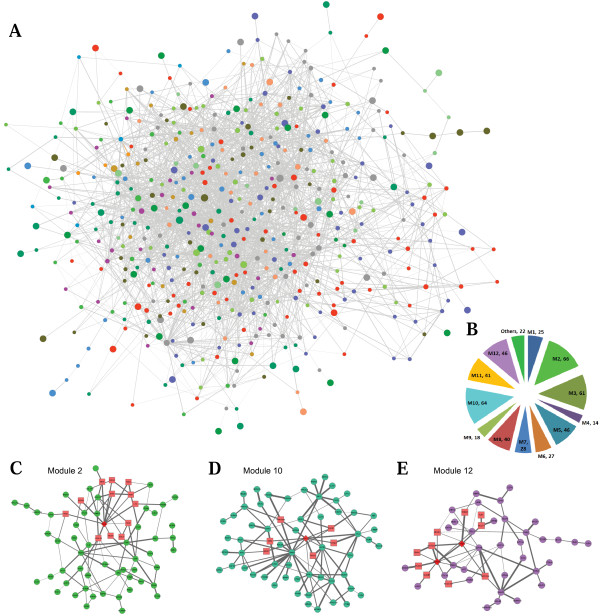
**CHD subnetwork and representative modules**. (A) CHD subnetwork extracted from the weighted PPI network. Different colors represent different modules. All the modules with size smaller than 10 are in the same green color. (B) Pie chart of module size, where modules with size smaller than 10 are grouped into category "others". (C-E) Three representative modules. Dark red diamonds represent putative disease genes, and red rectangles represent final candidate genes identified by our method.

Totally, our method resulted in a connected CHD subnetwork consisting of 498 nodes and 2413 edges (Figure 2A). We identified 12 major disjoint modules by assigning genes to the group which maximizes the information flow scores while keeping each module inter-connected the CHD subnetwork (Figure 2B-E). The size of each module is shown in Table 1 and the list of all genes in each module is shown in Additional File 2.

**Table 1:** Module information.

Module	Size	Interaction Score	Mutual Information (empirical *p*-value)	Correlation (*p*-value)	CHD genes in this module
M1	25	2.94E-01	2.80E-02	1.80E-03	GATA6

M2	66	4.33E-01	8.00E-03	3.62E-03	ELN

M3	61	2.83E-01	2.00E-03	9.82E-01	ACVR2B

M4	14	8.50E-02	2.70E-02	7.02E-02	MYH11

M5	46	1.47E-01	2.00E-03	1.67E-02	MYH7

M6	27	4.23E-01	1.80E-02	3.36E-03	CITED2

M7	28	3.44E-01	8.39E-01	5.76E-01	FLNA

M8	40	2.25E-01	<1.00E-06	3.66E-02	MYBPC3

M9	18	9.94E-02	7.40E-02	1.02E-02	GATA4, TBX5

M10	64	5.75E-01	<1.00E-06	8.95E-03	ACTC1

M11	41	4.32E-01	2.30E-02	4.80E-01	NKX2-5

M12	46	4.51E-01	2.00E-03	1.81E-05	NOTCH1, JAG1

Size means the number of genes in each module. Interaction score evaluates the topological relations between modules. Hub modules have higher scores while peripheral modules have lower scores. Mutual Information evaluates the significance of synergistic differential expression within a module compared with 1000 random gene sets of the same size. Correlation measures the significance of correlation coexpression of two interacting partners within a module compared with 1000 random edge lists of the same number.

### Modules as disease-markers

**Figure 3:**
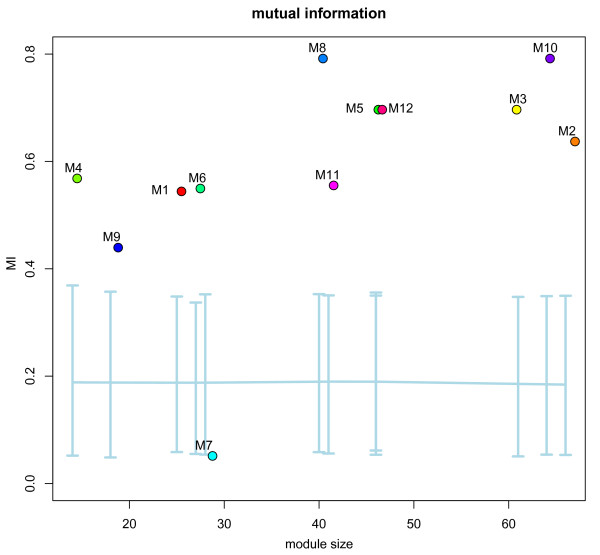
**Synergistic differential expression of modules compared with random gene sets of the same size**.

To quantitatively investigate the relationships of the identified modules with disease phenotype, we computed mutual information (MI) of each module with sample phenotype. The results are shown in Table 1. We used the microarray data, which represents mRNA extracted from right ventricles (RV) of 16 children with Tetralogy of Fallot (TOF, the most common type of CHD) at the time of reconstructive surgery with 5 controls (NCBI GEO Accession ID GSE26125), to compute the activity vector of each module across all 21 samples. We used MI as the discriminative score to assess whether each module activity vector has significant relationship with phenotypes. Among 12 modules, 10 have significant mutual information (empirical *p*-value<0.05) compared with 1000 random gene sets of the same size (shown in Figure 3). Most modules are significantly correlated with disease phenotype, reflecting the synergistic differential expression within them.

**Figure 4:**
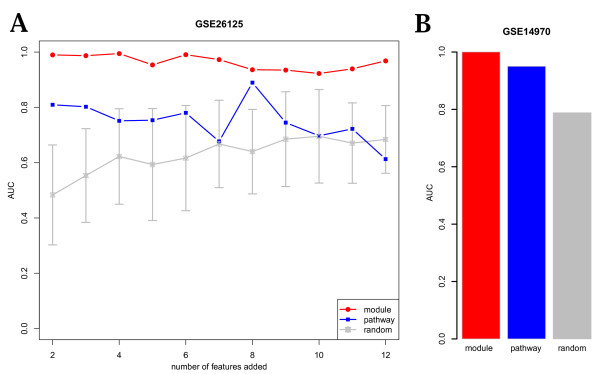
**Within- and cross-validation performance comparison of modules and pathways**. (A) Within-dataset classification evaluation. X-axis corresponds to the number of features added to the logistic classifier. Assuming that there are *K *features (modules/pathways/random gene sets), then for each *k *≤*K*, select the first *k *th modules to train the classifier. The final classification performance was reported as the AUC on the testing set using the classifier optimized from the validation set in a five-fold cross-validation. Modules were ordered in decreasing significance of MI, pathways were ordered in decreasing significance of enrichment, and random gene sets were in the order as their compared modules. (B) In cross-dataset classification evaluation, all 12 features were trained on GSE26125 and validated on 12 disease samples from GSE14970 combined with 5 controls from GSE26125.

To further evaluate the quality of our modules in terms of distinguishing disease from control samples, we computed the activity matrix of 12 modules (modules versus samples) and build logistic regression models [18] in a five-fold cross-validation approach. For comparison, we computed activity matrix of 12 KEGG pathways that are most enriched in the CHD subnetwork (Additional File 3). We also used 12 random gene sets of the same size as each module for control, and repeated this process 100 times. For experiments within the gene expression dataset of GSE26125, modules were first ranked by MI, and features were sequentially added to the classifier. Pathways were ranked by enrichment *p*-value, and were added sequentially. The result shows that modules are consistently better in the classification accuracy than pathways and random sets, while pathways are better than random sets only for the first five features (Figure 4A). To test the robustness of our module classifier, we also performed an independent cross-dataset experiment, in which classifiers were trained on GSE26125 and validated on GSE14970 (Figure 4B). In cross-dataset validation, both modules and pathways achieved similar high performance of classification accuracy, and clearly are much better than the random sets. Therefore, our modules are better classifiers of phenotype than pathways, and that their high classification performances are reproducible across independent microarray dataset. This provides evidence for the dysfunctional implication and importance of these identified modules.

### Within module coexpression and cross module coordination

**Figure 5:**
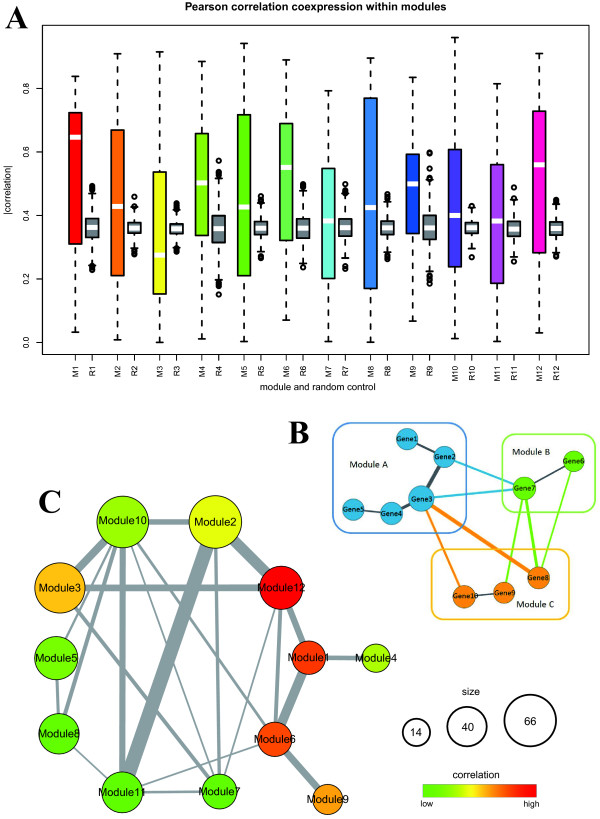
**Inter-module coexpression and intra-module coordination**. (A) To analyze coexpression within modules, we computed the average Pearson's correlation coefficient (PCC) of all edges in each module, and compared it with that of 1000 random control edge sets of the same size. (B) Module interaction identification was based on the weighted protein-protein interactions between modules. Edge width corresponds to the absolute value of PCC of two end nodes. Color edges represent cross-module interactions which will be used to compute module interaction, while grey edges are interactions within modules. (C) Intra-module coordination. Node colors range from green to red correspond to significance -*log*(*p*-value) of inter-module coexpression, node size corresponds to module size, and edge width corresponds to strength of interactions.

Besides comparing module expression profiles under disease and control status to evaluate its synergistic differential expression, another way to utilize microarray data is to exploit gene coexpression relationships within modules and across modules. Under the well-accepted hypothesis that genes exhibiting similar expression patterns across sample status are likely to have functional relevance [19], we reasoned whether genes within modules are significantly coexpressed, and whether modules have intense interactions in terms of interacting partners that are highly coexpressed. To analyze coexpression within modules, we computed the average Pearson's correlation coefficient (PCC) of all interacting proteins in each module, and compared it with that of 1000 random selected protein pairs of the same size (Figure 5A, Table 1). Result shows that 9 modules have significant coexpression (Mann-Whitney test, *p*-value<0.05), and all of them also have significant MI at the same time. If all 12 modules are regarded as a whole and compared with 12 random gene sets, the result is also significant (Mann-Whitney test, *p*-value = 2.96e-06).

To analyze intra-modular connectivity, we detected the interaction among disjoint modules based on weighted protein interactions (Figure 5B). Module interaction network is visualized in Figure 5C, where only the 22 significant interactions (corrected *p*-value<0.05) are shown. For each module, we defined its intra-modular connectivity as the sum of the absolute value of correlation for all its outgoing edges, and used this score to evaluate its network centrality (Table 1). Module interaction network not only reveals connectivity patterns between modules and their positions in CHD subnetwork, but also sheds light on the implications of various biological processes and pathways represented by each module. Therefore, besides topological meanings of this score, we would identify its biological underpinnings after examining the compositions of modules using annotated gene ontologies in the next subsection.

### Functional enrichment analysis of modules reveals crucial biological processes of CHD

Utilizing our previously developed NOA (Network Ontology Analysis) method of functional enrichment analysis [20], we identified enriched GO terms in each module. The representative enriched GO terms with FDR<0.05 in each module are presented in Table 2, and the list of all enriched GO biological processes are in Additional File 4. The top-scored Module 10 (Figure 2D) is enriched in anatomical structure morphogenesis, cytoskeleton organization and cellular component assembly. Since CHD manifests itself through various aberrations in heart morphogenesis, and more than half of known causal disease genes are transcriptional regulators of heart morphogenesis, the fact that anatomical structure morphogenesis ranks 1^st ^in the most important module is expected. The other two categories, i.e. cytoskeleton organization and cellular component assembly, can contain downstream effector genes involved in muscle development, such as sarcomerec gene like ACTC1, DYNLL1 and DMD. Particularly, ACTC1 is a known causal gene in Module 10 [21,22]. It encodes the cardiac actin protein that is an essential structural component of the thin filaments of sarcomeres. Mutations in ACTC1 seem to reduce affinity of actin for myosin, and cause various CHD phenotypes such as ASD (atrial septal defect) and VSD (ventricular septal defect) [22]. ACTC1 contains all GO terms significantly enriched (*p*-value<0.05) in Module 10, demonstrating the functional relevance of Module 10 to CHD. Module 12 (Figure 2E) ranks 2^nd^, and is enriched in negative regulation of apoptosis, programmed cell death, cell differentiation and developmental process. Module 12 is also highly enriched in Notch signaling pathway, which plays roles in various developmental processes by controlling cell fate decisions, and is known to be very important in cardiac development. Module 12 contains 2 causal genes, NOTCH1 [23] and JAG1 [24], again, both of which participate in all of the 40 top enriched GO processes (*p*-value<1.6E-4). Table 2 also suggests that in each module, CHD genes participate in most of its top ranked GO terms, and the functional of the module is very similar to its contained CHD genes. This can also prove the functional coherence within each module, and functional correlation between these modules.

**Table 2:** Enriched GO terms in each module.

Module	GO ID	p-value	GO description	CHD genes in this module and with this GO term	FDR
M1	GO:0019219	8.00E-10	regulation of nucleobase, nucleoside, nucleotide and nucleic acid metabolic process	GATA6	1.90E-07
	
	GO:0051171	1.10E-09	regulation of nitrogen compound metabolic process	GATA6	2.70E-07
	
	GO:0051716	4.00E-08	cellular response to stimulus	GATA6	9.70E-06
	
	GO:0045941	3.20E-07	positive regulation of transcription	GATA6	7.80E-05

M3	GO:0007178	7.30E-34	transmembrane receptor protein serine/threonine kinase signaling pathway	ACVR2B	2.70E-31
	
	GO:0007179	3.70E-23	Transforming growth factor beta receptor signaling pathway		1.40E-20
	
	GO:0032925	1.70E-12	regulation of activin receptor signaling pathway	ACVR2B	6.60E-10
	
	GO:0045597	1.10E-09	positive regulation of cell differentiation	ACVR2B	4.40E-07
	
	GO:0051239	5.60E-09	regulation of multicellular organismal process	ACVR2B	2.10E-06
	
	GO:0010646	1.60E-08	regulation of cell communication	ACVR2B	6.40E-06

M4	GO:0006468	1.20E-04	protein amino acid phosphorylation		5.80E-03

M5	GO:0006259	7.60E-05	DNA metabolic process		1.78E-02

M6	GO:0045941	4.50E-06	positive regulation of transcription	CITED2	5.90E-04
	
	GO:0051254	9.80E-06	positive regulation of RNA metabolic process	CITED2	1.20E-03
	
	GO:0045935	1.00E-05	positive regulation of nucleobase, nucleoside, nucleotide and nucleic acid metabolic process	CITED2	1.30E-03

M7	GO:0035556	2.20E-04	intracellular signal transduction	FLNA	2.52E-02

M8	GO:0006936	1.90E-08	muscle contraction	MYBPC3	3.00E-06
	
	GO:0003008	1.80E-06	system process	MYBPC3	2.90E-04
	
	GO:0007010	8.20E-06	cytoskeleton organization		1.30E-03
	
	GO:0030036	8.10E-05	Actin cytoskeleton organization		1.31E-02

M9	GO:0007154	9.40E-06	cell communication	GATA4, TBX5	1.40E-03
	
	GO:0048545	7.50E-05	response to steroid hormone stimulus	GATA4	1.15E-02
	
	GO:0006629	2.30E-04	lipid metabolic process		3.57E-02

M10	GO:0022607	5.10E-06	cellular component assembly	ACTC1	7.30E-04
	
	GO:0009653	9.30E-05	anatomical structure morphogenesis	ACTC1	0.0132

M11	GO:0031334	2.20E-04	positive regulation of protein complex assembly	NKX2-5, SRF	4.42E-02

M12	GO:0043066	2.20E-12	negative regulation of apoptosis	NOTCH1	6.20E-10
	
	GO:0045595	1.90E-10	regulation of cell differentiation	JAG1, NOTCH1	5.50E-08
	
	GO:0051093	1.90E-09	negative regulation of developmental process	JAG1, NOTCH1	5.40E-07
	
	GO:0045596	9.70E-09	negative regulation of cell differentiation	JAG1, NOTCH1	2.70E-06
	
	GO:0007219	7.30E-08	Notch signaling pathway	JAG1, NOTCH1	2.00E-05

CHD genes participate in most of the top enriched GO processes in each module and the function of a module is very similar to those of the putative disease genes in it. Only GO terms with FDR<0.05 are shown below.

We have shown that modules with highest scores, being in the central place of the subnetwork, are enriched in core processes related to heart development, and that CHD genes participate in all the top ranked GO processes in a module. We then investigated the modules with lowest scores in the marginal place of subnetwork and identified their relations with CHD. Module 4 (Additional File 5) is ranked as the last, and is enriched in phosphorus metabolic process. Module 9 (Additional File 5) is enriched in cell communication, response to steroid hormone stimulus, lipid metabolic process and positive regulation of transcription. Module 5 (Additional File 5) is enriched in regulation of macromolecule biosynthetic process and nitrogen compound metabolic process. Although these GO processes seem to have less relationship with CHD, these auxiliary roles are important to facilitate key processes of heart development. Considering the fact that fetal heart developmental program involves intense transcription regulation, ligand-receptor interactions and signaling transduction, various macromolecules including hormones, cytokines and growth factors in the circulation or in the extracellular space of the heart, acting as ligands, can stimulate receptors in the cell membrane of cardiac cell [2], it is reasonable to assume that cell communication, transcription regulation, steroid hormone stimulus and macromolecules metabolism are more generally associated with CHD and can actually facilitate all related processes. Since they are not specific to CHD, they appear on the marginal place of the subnetwork. The fact that these modules can also contain causal disease genes demonstrates their relevance to CHD. For example, GATA4 and TBX5 are in Module 9, and both of them are important transcription factors in developmental processes. TBX5 protein associates with cardiac transcription factors including GATA4 and NKX2-5, and they activate many downstream cardiac effector targets such as sarcomeric proteins and vasoactive proteins. Various kinds of mutations in GATA4 and TBX5 can lead to various subtypes of CHD [25,26].

### Module-pathway crosstalk analysis reveals hub modules regulating key pathways of CHD

**Figure 6:**
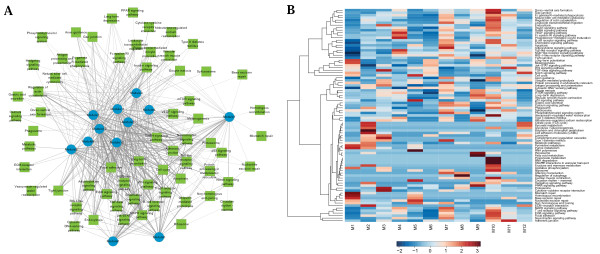
**Module-pathway crosstalk**. In both subfigures, only pathways which contain at least one gene on the CHD subnetwork are shown. (A) Network view of module-pathway crosstalk. Blue circles represent modules and green rectangles represent pathways. Edge width corresponds to strength of interaction. (B) Heatmap of module-pathway crosstalk. For clarity, module-pathway activity matrix is x-scaled, which means, for each pathway, colors ranging from blue to red represent the influential power of one particular module compared with all 12 modules.

To analyze pathways represented by modules, and how they are coordinated by modules to account for the observed phenotypes, we went beyond identifying lists of pathways significantly enriched in each module, or simply counting the number of overlapping genes shared by the module and the pathway. As previously shown, there is a distinction between a pathway and a module. A pathway is a specific information-flow conduit, consisting of a series of molecular interactions while a module is an information-processing unit with self-contained cellular functions [17]. Therefore, modules can contain multiple pathways while pathways can be coordinated by various modules to allow inter-module connections. With this in mind, we implemented an analysis of module-pathway crosstalk similar to previous module interaction procedure. For a given module-pathway pair, we considered both common genes and weighted protein-protein interactions between them (See Methods for details of constructing module-pathway crosstalk network). Network view of module-pathway crosstalk is shown in Figure 6A, where circles represent modules, rectangles represent pathways, and edge width corresponds to strength of interaction between module and pathway. Heatmap of pathway-module crosstalk is shown in Figure 6B, where rows represent 85 pathways that are significantly (empirical *p*-value<0.01) influenced by at least one module, and columns represent modules and their influence to the 85 pathways. Module 10 is the most influential and many of its influenced pathways are closely related to heart progression, such as cardiac muscle contraction, Dorso-ventral axis formation, gap junction and regulation of actin cytoskeleton. Specifically, the gap junction is very important in cardiac muscle, through which the signal to contract is efficiently passed, allowing the heart muscle cells to contract in tandem. For example, GJA1 encodes a gap junction protein, and gene conversion in GJA1 has been found in patients with CHD [27]. In brief, these influenced pathways are consistent with major roles of Module 10 in biological processes like anatomical structure morphogenesis and cytoskeleton organization. Module 12 is the only one that can influence Notch signaling pathway, which is also consistent with its GO enrichment. Module 12 and module 2 are the only two modules that regulate ECM (extracellular matrix)-receptor interaction, a very important pathway in tissue and organ morphogenesis and in the maintenance of cell and tissue structure and function. The extracellular matrix protein, FLNA, for example, can cross-links actin filaments and participates in anchoring actin cytoskeleton to membrane proteins. Loss-of-functions mutations in FLNA are lethal to male and cause various CHD-related phenotypes in female [28,29]. Although Module 12 and Module 2 do not affect as many pathways as Module 10 does, their dominant roles in regulating two essential pathways demonstrate their substantial importance during heart development. From Figure 6B, Module 7 also seems important because it interacts with many pathways, while we find that it has much less influence to pathways highly related to CHD. Compared with Module 10, it shows significant drop for many essential pathways related to CHD such as cardiac muscle contraction, Dorso-ventral axis formation, gap junction and regulation of actin cytoskeleton, while some pathways without direct relations to CHD such as Type I diabetes mellitus and Olfactory transduction, exhibit significant increase. Therefore, we reasoned that the importance of a module should be evaluated not by the number of pathway that it affected, but by its influence to important pathways implicated in CHD.

### Prioritization of candidate disease genes by module analysis

We have proved the robustness of our modules for disease phenotype classification, investigated their functional roles during heart development, their implications in CHD, and their interactions with each other as well as with those documented pathways. We also provided an application of identified dysfunctional modules to detect disease genes. We brought out a list of prioritized candidate disease genes by integrating module memberships, network centrality analysis and GO semantic similarities (See Additional File 6 for detailed methods). We first identified candidate genes that are either on the shortest paths connecting 14 causal disease genes or the first interacting neighbors of them in the CHD subnetwork, then assigned a score to each of them with investigation of its module membership and its GO semantic similarity with the putative disease genes in the same module, and at last filtered genes with significant different network locations (Additional File 6), which resulted in a final list of 60 candidate disease genes. The top ten candidate genes are shown in Table 3 and the full list of candidate disease genes are shown in Additional File 6.

**Table 3:** Top 10 candidate disease genes and supporting evidence.

Gene	Score	Description (GeneCards Version 3)	Supporting evidence	PubMed ID
HAND2	6.87	heart- and neural crest derivatives-expressed protein 2, essential for cardiac morphogenesis, particularly for the formation of the right ventricle and of the aortic arch arteries	population study: various mutations found in TOF patients	20819618

FOS	4.09	Nuclear phosphoprotein which interacts JUN/AP-1 transcription factor. Has a critical function in regulating the development of cells destined to form and maintain the skeleton.	literature text-mining: cardiac hypertrophy	16696897, 10328763, 12713689, 16259952

NOTCH2	2.5	Functions as a receptor for membrane-bound ligands Jagged1, Jagged2 and Delta1 to regulate cell-fate determination.	population study: various mutations found in CHD patients	16773578

MLLT4	1.72	Belongs to an adhesion system which plays a role in the organization of cell-cell adherens junctions (AJs). Nectin- and actin-filament-binding protein that connects nectin to the actin cytoskeleton	similar functions to FLNA [28,29]: actin cytoskeleton, and to GJA1 [27]: cell-cell adhensions	see reference of supporting evidence

THBS1	1.7	Adhesive glycoprotein that mediates cell-to-cell and cell-to-matrix interactions	genetic association database: myocardial infarct	12482844, 16684956

MAPK14	1.69	act as an integration point for multiple biochemical signals, and are involved in a wide variety of cellular processes such as proliferation, differentiation, transcription regulation and development.	literature text-mining: pulmonary disease chronic obstructive	19880675, 17959643, 20093202, 19004925

ELK1	1.56	Can form a ternary complex with the serum response factor and the ETS and SRF motifs of the fos serum response element	SRF [4] is a cardiac-tissue enriched transcription factor that regulate many cardiac effector genes during embryogenesis	see reference of supporting evidence

MAPKAPK5	1.54	similar to MAPK14		

DCN	1.52	This protein is a component of connective tissue, binds to type I collagen fibrils, and plays a role in matrix assembly.	literature text-mining: myocardial infarction, heart failure, congenital malformation, vascular diseases	17558846, 9162605, 18514055, 9493904

NUMB	1.49	plays a role in the determination of cell fates during development. associate with disease gene NOTCH1	MGI database: targeted knock-out in mice affect cardiovascular systems	11412999

Score is a candidate's GO semantic similarity with disease genes in the same module, and IEA GO terms are excluded. Description briefly introduces the candidate's CHD-related functions. Supporting evidence lists the types of literature support, and PubMed IDs of related articles are provided in the last column for reference.

HAND2 has significantly higher score than other candidate genes and ranks 1^st ^in our list. HAND2, and causal disease gene HAND1, whose somatic mutations have been reported to contribute to CHD [30], both belong to basic helix-loop-helix (bHLH) transcription factors, and are expressed in cardiac mesoderm during embryogenesis. HAND1 expression is limited in future left ventricle while HAND2 expression is limited in future right ventricle [31]. Targeted gene deletion of HAND2 in mouse embryos resulted in embryonic lethality from heart failure [32]. In a recent research, Shen et al. [33] screened for mutations in the HAND2 genes in 131 patients with various forms of CHD. Seven mutations in HAND2 were identified in 12 out of these patients. It is very likely that HAND2 is indeed a CHD disease gene. FOS is ranked 2^nd^, and can form transcription factor complex AP-1 with proteins from the JUN family. FOS proteins are implicated as regulators of cell proliferation, differentiation and transformation. Literature text-mining using GeneCards Version 3 [34] reveals that FOS is involved in cardiac hypertrophy. NOTCH2 ranked 3^rd^, and together with NOTCH1, whose autosomal dominant mutations can cause CHD [23], belong to single-pass transmembrane receptors that regulate many developmental pathways. Mutations in gene encoding NOTCH2 leads to Alagille syndrome [35], with cardiac phenotypes of peripheral pulmonary artery stenosis and septal defects. Besides genes with high score and well supported by literature search, our candidate gene list also contains genes with low scores. Currently, these genes have fewer, if any, favorable GO evidence similar to disease genes in the module, but some of them would be potential novel disease genes and worth further experimental research.

## Discussion

It has long been noted that genetic, physical interaction and transcriptional expression data are complementary to each other in response to molecular perturbations [36-38]. We also found that not all CHD genes are differentially expressed and that none of them can be the target gene. Therefore, using a source-sink relationship is better than simply using disease genes as seeds for module identification in that the former regards the whole transcritome as phenotype data while the later merely utilizes disease/control status as phenotype. Actually, it was pointed out previously that a gene-disease phenotype association provides little insight into the molecular mechanism for the association [10,39]. Bridging the discordance between CHD genes and target genes through a source-sink approach can not only identify intermediary genes not discovered in the transcriptional data itself, but also reveal dysfunctional or even causal pathways and modules for CHD. In our approach, information from the source gene will propagate its effect via protein-protein interactions, and DE genes which cover the majority of patients represent common dysregulated pathways in CHD. It is reasoned the common pathways, regardless of various kinds of source mutations, are responsible for the final set of similar CHD phenotypes. One of the applications of methods like ours is that by identifying intermediary genes involved in signal transduction, it can suggest potential intervention nodes for drug targeting.

Our method simulated the causality-response relationship between the known disease genes and differentially expressed genes. The dysfunctional information transferring between them in the network is utilized to identify the disease modules, which have proved to be highly related to the phenotypes. The robust performance has to be evaluated for our approach in computing information score -- we identify the shortest paths from a source gene to all target genes, and compute information score in this subnetwork, which means that the information score for each gene in this subnetwork is only related to a particular source gene. The choice of subsets of source genes changes available subnetworks, and can influence the result during the module identification procedure, in which a gene is reassigned to the modules which maximize its information score while maintaining connectivity. To evaluate the robustness and reproducibility of modules when some known disease genes are withheld (Additional File 6), we generated 100 alternative sets of testing modules and computed the overlap with the reference modules. The modules have significantly consistent overlap with testing modules (Additional File 6). This indicates that our methods are robust to identify these modules when we eliminate some source genes. The details of the tests and results are available in Additional File 6. From these dysfunctional modules, we also identified some candidate CHD genes which are based on the score of associating with the causal disease genes.

## Conclusions

Using a network-based approach, we identify dysfunctional modules and disease genes in CHD by modeling the information flow from source disease genes to targets of differentially expressed genes. Several other studies also inferred causal genes, dysregulated pathways and central nodes of a network using flow-based approach [11,15,38]. However, we went a step further and brought a group of dysfunctional modules. By considering only one source node and multiple target nodes in each circuit flow instance, and iterating over all source nodes, we identified 12 dysfunctional modules which are well validated by various types of statistical measures, an independent dataset and biological annotations. Although not required by our method, each module contains CHD genes, making it possible to prioritize candidate CHD genes using a "guilt-by-association" approach. The identified dysfunctional modules show their biomarker properties for CHD phenotypes and these candidate disease genes will benefit further research.

## Methods

### Datasets

GSE26125 consists of RNA extracted from right ventricle of 16 idiopathic TOF (Tetralogy of Fallot) patients and 5 controls obtained at the time of reconstructive surgery. The microarrays were CodeLink Human Whole Genome Bioarray, which contain > 54,000 probes. Raw data was preprocessed as described in the original paper [8,9]. The microarrays of GSE14970 were Affymetrix Human Genome U133 Plus 2.0 Array. Raw data was preprocessed using RMA algorithm in R bioconductor [40]. Probe sets were mapped to NCBI entrez genes, and for genes with multiple probe sets, the average expression value of all corresponding probe sets was used. Together 8547 genes were expressed in the right ventricle tissues on GSE26125, which will be used for our study. 6818 genes were expressed in both GSE26125 and GSE14790, whose expression values will be used for cross-data validation. GSE14970 consists of 12 diseased samples (5 pre-operation acyanotic TOF and 7 pre-operation cyanotic TOF in the RV tissue) without controls but is the only alternative CHD-related dataset at this time, we added 5 control samples from GSE26125 and z-transformed the combined dataset.

### Conditional-specific interaction network

We utilized protein-protein interaction data from several curated interaction databases in human, i.e., HPRD, BIND, BioGrid, IntAct, and MINT [41-45]. Those interactions contained in at least three of the five databases were selected. Only genes both in the PPI network and in the microarray were used in the following study, and we extracted the maximum connected component of the network, which consists of 4761 nodes and 18084 edges. To construct a context-specific protein interaction network, the weight of each interaction between protein *u *and *v*, as well as the conductance of each edge *e(u,v)*, was defined as their correlation value, i.e. *w*(*u*,*v*) = |*corr*(*u*,*v*)|

### Module identification

Source CHD genes were extracted from a recent literature review [2], and 14 of them were mapped to the conditional-specific protein interaction network. Similar to the identification of Differentially Expressed (DE) genes and target genes in previous work [11], we have scaled and centered gene expression values into a Z-score. A gene is defined to be a differentially expressed gene in a disease case if its normalized gene expression value has a p-value <0.01 in the given disease case versus all controls using t-test, and a gene is defined as a target gene, if it has p-value<0.01 in more than 75% disease cases. Based on the definition of information flow score in previous work [15], we computed the information flow score of node *k *for a given source gene *s *as the sum of current through node *k *among all pair-wise combinations of the source *s *and all target DE genes *Tar*, detailed rationales for this information flow score can be referred to the original paper [15]. There are together 14 source genes, and the information flow through node *k *for a given source *s *is

$$I_{k}^{}=\frac{1}{2}\sum_{t\in Tar}\left(\sum_{Nei}^{}\left|I_{kNei}\right|\right),$$

where *I_kNei _*is the current between node *k *and its neighbor, ∑*_Nei _*is the sum of all neighbors connected to node *k*.

For a given pair of source node *s *and a target node *t*(*t*∈*Tar*), we have the following equation for node *k*(*k*≠*t*) according to Kirchhoff's current law:

$$\frac{\sum_{Nei}\left(v_{Nei}-v_{k}\right)}{R_{kNei}}+I_{k}=X,$$

where *v_k _*is the voltage at node k, and the sum ∑*_Nei _*is for all direct neighbors of node *k*. *X *is a unit value of current when *k = s *and 0 otherwise. Let *G=(N, E) *represent the protein interaction network where *N *is the set of nodes and *E *is the set of edges, node voltages *V *can be computed by the following linear system of equations according to Ohm's law:

P×V=I,

where P is a symmetric (N-1)×(N-1) conductance matrix, V is a (N-1)×1 vector of node voltages and *I *is a (N-1)×1 vector of currents passing through the nodes, the row and column of ground node is removed since its voltage is zero.

Such a linear system considered all interactions in the global network and required that every interaction can have a regulatory role for the expression of the target gene. We therefore implemented a straightforward heuristic approach: we identified the nodes that are on the maximum weighted shortest paths from a source gene to all target genes and performed the calculations of information flow score on this subnetwork. The modules were identified using the following algorithms.

Pesudocode for identifying modules is shown below:

1. For each causal disease gene s_i_,

a. For each target DE gene *t_j_*,

i. identify all shortest paths from s_i _to *t_j_*,

ii. choose the path with maximum sum of weight,

b. merge all nodes on the chosen paths into a subnetwork *Sub_i_*, for s_i_,

c. compute the information flow score *I_k _*for each node in *Sub_i_*.

2. For each gene on the subnetworks, put it into the module *M_m _*where its information flow score is maximum,

3. For each module *M_m_*, identify the unconnected genes. After extracting the unconnected genes from all modules, simultaneously put each of them into another module *M_n _*where the information score is the second largest.

4. Repeat step 3 until all modules are interconnected.

5. For genes that cannot connect to any module in which it has an information flow score, regard each of their connected components as a new module.

The source code for module identification and related data in this study can be downloaded at http://doc.aporc.org/wiki/CHD.

### Mutual information

For each of the identified dysfunctional modules, we quantified its discriminative power by an information-theoretic scheme [13]. Various methods have been developed to infer expression activity from a group of functionally related genes. Just as reasoned in previous research [46] that not all genes within a pathway are highly discriminative and that using a subset of "condition-responsive genes" achieves better discriminative power than conventional gene and pathway based approach, we defined the differentially expressed (t-test, corrected *p*-value<0.05) genes as those that can represent the synergistic activity of a module or a pathway. For these differentially expressed genes, let *a*(*M*) denote the preprocessed and z-transformed m-dimensional microarray expression vector across 16 disease and 5 control samples on GSE26125. Let *c *denote an m dimensional binary vector representing the phenotype class of each sample, and *c_i _*= 1 if the ith sample is disease, 0 if it is control. The aggregated expression activity vector *a*(*M*) for the module induced by its differentially expressed genes is

$$a\left(M\right)=\overset{k}{\underset{g\in M}{\sum }}\frac{a\left(g\right)}{\sqrt{k}}.$$

We defined the discriminative score *S*(*M*) as *MI *(*a*'(*M*),*c*), and the mutual information MI between *a*', a discretized form of *a*, and *c *as

$$S\left(M\right)=MI\left(a^{\prime }\left(M\right),c\right)=\underset{x\in a^{\prime }}{\sum }\overset{}{\underset{y\in c}{\sum }}p\left(x,y\right)\log\frac{p\left(x,y\right)}{p\left(x\right)p\left(y\right)}.$$

Here, *a*' is obtained by discretizing *a *into [log2(# of samples)+1] = 6 equally spaced bins [13], and the midpoint of each bin is used as the value for *a*'. × and y enumerate values of *a *and *c*, p(x,y) is the joint probability density function (pdf) of *a*' and *c*, and p(x) and p(y) are the marginal pdf's of *a *and *c*.

To get empirical *p*-value for the discriminative score, we also generated *N *random gene sets of the same size as each module *R*, aggregated their genes' expression vectors *a*(*R*) and computed their MI with phenotype *S*(*R*):

$$\text{empirical }p\text{ - value = }\frac{\left(\text{the number of }S\left(R\right)>S\left(M\right)\right)}{N},$$

where *N *is set as *N *= 1000.

### Phenotype classification comparison

To evaluate the ability of our modules for discriminating phenotypes, we performed both within-dataset and cross-dataset classification validation. To allow for comparison with pathways and random controls, we aggregated the expression values of DE genes in each module and in each pathway from the CHD subnetwork. We also aggregated the expression of random gene sets with the same size as the module. The activity vector aggregation method is the same as in the former Mutual Information section. Modules were ordered in decreasing significance of MI, pathways were ordered in decreasing significance of enrichment and random gene sets were ordered the same sequence as modules. Then, logistic regression models were trained on the module activity matrix (modules versus samples), pathway activity matrix (pathways versus samples) and random gene sets (gene sets versus samples). For within-dataset experiments, each time 4/5 of the samples were implemented as the training set to build the classifier and the remaining 1/5 samples were used as testing set to evaluate the performance (five-fold cross validation). Assuming that there are *K *modules, then for each *k *≤ *K*, select the first *k*_th _modules to train the classifier. Pathways and random gene sets were also used in similar manner. The final classification performance was reported as the Area Under ROC Curve (AUC [47]) on the testing set using the classifier optimized from the validation set. For cross-dataset experiment, all 12 modules/pathways/random gene sets were trained on GSE26125 and validated on 12 disease samples from GSE14970 combined with 5 controls from GSE26125. All preprocessed microarray expressions were *z*-transformed before activity vector is aggregated. For both within- and cross-dataset experiments, random controls were repeated 100 times.

### Correlation and crosstalk

Previous work [48] has identified crosstalk of pathways based on overlapping genes and weighted interactions, we used similar metrics to measure module coordination and module-pathway crosstalk in the present study. For each module-module pair *X *and *Y*, we firstly identified the edges with two end nodes, each of which belongs to one module, and then we summed up the weights of all these connecting edges using

$$score\left(X,Y\right)=\overset{}{\underset{x\in X}{\sum }}\overset{}{\underset{y\in Y}{\sum }}\left|weight\left(x,y\right)\right|.$$

For module-pathway crosstalk, we only considered the pathway which contains at least one gene on the CHD subnetwork in order to filter out unspecific pathways. For each module-pathway pair *M *and *P*, we firstly identified the edges with two end nodes, each belonging to the module and the pathway, and detected the nodes in both the module and the pathway. Then we summed up the weights edges and number of nodes using

$$score\left(M,P\right)=\frac{\sum_{x\in M,y\in P}\left|weight\left(e\left(x,y\right)\right)\right|+\overset{}{\underset{x\in M\cap P}{\sum }}1}{\sqrt{\left|M\right|}\sqrt{\left|P\right|}}.$$

where ∑*_x_*_∈_*_M_*,_y∈*P*_|*weight*(*e*(*x*,*y*))| corresponds to the sum of the weight of all edges, and {*e*(*x*,*y*):*x*∈*M*,*y*∈*P*} are those edges that connect a pathway *P *and a module *M*. $\overset{}{\underset{x\in M\cap P}{\sum }}1$ corresponds to the total number of common nodes shared by *M *and *P*, where each node is given the weight equals to 1. $\sqrt{\left|M\right|}\sqrt{\left|P\right|}$ corresponds to the square root product of the number of nodes in module *M *and pathway *P*. The summation is such divided in order to normalize the size of module and pathway.

To get empirical *p*-value, we generated 1000 gene sets pairs containing the same number of nodes as the module-module or module-pathway pair, and computed their interaction scores. Random controls were generated from genes on the whole weighted PPI network, and genes in the KEGG. The significance of interaction is defined as

$$\text{empirical }p\text{ - value = }\frac{\left(\text{the number of }S\left(R\right)>S\left(M\right)\right)}{N2},$$

where *N*_2 _is set as *N*_2 _= 1000.

## Competing interests

The authors declare that they have no competing interests.

## Authors' contributions

D.H., Z.P.L. and L.C. conceived the research, Z.P.L. and D.H. designed the framework and experiments, D.H. and Z.P.L. performed the experiments, D.H. and Z.P.L. drafted the manuscript, L.C. revised the manuscript and supervised the project. All authors read and approved the final manuscript.

## Supplementary Material

Additional file 1 **List of selected target genes and sample distance of 21 expression profiles**. Click here for file

Additional file 2 **Genes in each module**. Click here for file

Additional file 3 **Pathways enriched in CHD subnetwork using hypergeometric test**. Top 12 pathways are used for classification evaluation. Click here for file

Additional file 4 **List of enriched GO terms in each module**. Click here for file

Additional file 5 **Network view of Modules 4, 5 and 9**. Click here for file

Additional file 6 **Details of prioritizing of candidate disease genes by module analysis and robustness analysis of identified modules**. Click here for file
