# Supplementary material for: Identification of dysfunctional modules and disease genes in congenital heart disease by a network-based approach
Source: BMC Genomics. 2011 Dec 2;12:592. doi: 10.1186/1471-2164-12-592 (PMC3256240; doi:10.1186/1471-2164-12-592)
Supplement: Additional file 1 — List of selected target genes and sample distance of 21 expression profiles. [file 1471-2164-12-592-S1.DOC]

### Additional File 1: List of selected target genes and sample distance of 21 expression profiles.

**Table A1.1: List of selected target genes.**

| **Target Gene** |
| --- |
| MSH2 |
| CBX8 |
| SLC3A2 |
| SEC63 |
| PRUNE |
| ASAH1 |
| CREM |
| DLAT |
| PSMA1 |
| ALDOA |
| KCNMA1 |
| NISCH |
| NCAM2 |
| SUCLA2 |
| BSDC1 |
| HINT1 |
| F3 |
| ANXA7 |
| CLTA |
| FNTA |
| KIR2DL4 |
| TAF15 |
| SFRS11 |
| TRAP1 |
| ATPAF1 |
| VPS29 |
| CMYA5 |
| ZNF512B |
| EPO |
| MTHFD2 |
| PRMT1 |
| RNF2 |
| CCL7 |
| HBXIP |
| TNFAIP8 |
| ALS2CL |
| FNDC5 |
| OTUD4 |
| FBXO22 |
| ZFYVE9 |
| COPS5 |
| RPS27L |
| TRIM3 |
| WDR42A |
| ANPEP |
| CAD |
| ARL2 |
| BZRAP1 |
| GABARAPL2 |
| NRGN |
| ANKS1B |
| DAD1 |
| ACAA2 |
| SPRY4 |
| BMP4 |
| PPP2R2A |
| CYBB |
| HMGN1 |
| INPPL1 |
| SOD2 |
| VDAC1 |
| CNTNAP1 |
| SNAP29 |
| WWP1 |
| UNC13D |
| BRP44L |
| ACAT1 |
| RAB21 |
| REXO2 |
| CRH |
| INSIG2 |
| VTA1 |
| ZNF44 |
| SOX10 |
| B2M |
| EPHB6 |
| EIF1AX |
| MRPL12 |
| MAPK8IP3 |
| VPS24 |
| NDUFV1 |

**Figure A1.1.** **Sample distance of 21 expression profiles.**
