# Supplementary material for: Identification of dysfunctional modules and disease genes in congenital heart disease by a network-based approach
Source: BMC Genomics. 2011 Dec 2;12:592. doi: 10.1186/1471-2164-12-592 (PMC3256240; doi:10.1186/1471-2164-12-592)
Supplement: Additional file 2 — Genes in each module. [file 1471-2164-12-592-S2.DOC]

### Additional File 2: Genes in each module.

**Table A2.1. Genes in each module.**

| **Module** | **Gene** | **Current flow** |
| --- | --- | --- |
| Module1 | MSH2 | 541.62 |
| Module1 | YWHAQ | 1671.8 |
| Module1 | CBX8 | 0 |
| Module1 | NME1 | 547.65 |
| Module1 | SLC3A2 | 153.76 |
| Module1 | SEC63 | 42.091 |
| Module1 | PRUNE | 0 |
| Module1 | APEX1 | 1633.9 |
| Module1 | DDX5 | 1747.2 |
| Module1 | COPS6 | 1318.2 |
| Module1 | EP300 | 5085.9 |
| Module1 | CREB1 | 1245.7 |
| Module1 | GSTK1 | 1541.4 |
| Module1 | MSH6 | 968.34 |
| Module1 | NCOA3 | 836.81 |
| Module1 | NBN | 562.53 |
| Module1 | RAD23A | 387.02 |
| Module1 | THRA | 618.43 |
| Module1 | CRIP2 | 352.32 |
| Module1 | CTBP2 | 171.02 |
| Module1 | ELK1 | 412.67 |
| Module1 | GATA6 | 27.095 |
| Module1 | MDM4 | 650.7 |
| Module1 | PRKG1 | 487.14 |
| Module1 | KLF2 | 87.422 |
| Module2 | AKT1 | 3115.7 |
| Module2 | CSNK2A2 | 1946.6 |
| Module2 | VIM | 1870.4 |
| Module2 | RNPS1 | 1055.5 |
| Module2 | ALAS2 | 213 |
| Module2 | ASAH1 | 138.91 |
| Module2 | CREM | 411.25 |
| Module2 | DLAT | 0 |
| Module2 | PHC2 | 867.58 |
| Module2 | PRNP | 756.79 |
| Module2 | PSMA1 | 1070.7 |
| Module2 | ALDOA | 343.75 |
| Module2 | KCNMA1 | 0 |
| Module2 | PDK2 | 213 |
| Module2 | RABGGTB | 844 |
| Module2 | NISCH | 5.893 |
| Module2 | PNKP | 329.65 |
| Module2 | TRIM29 | 213 |
| Module2 | NCAM2 | 0 |
| Module2 | SUCLA2 | 0 |
| Module2 | BSDC1 | 0 |
| Module2 | HINT1 | 0 |
| Module2 | ARAF | 2093.7 |
| Module2 | MAP2K1 | 1187.8 |
| Module2 | MMP2 | 1049.4 |
| Module2 | YWHAZ | 1376.7 |
| Module2 | SETDB1 | 1848.4 |
| Module2 | HMOX2 | 876.47 |
| Module2 | ILK | 1582.9 |
| Module2 | TGM2 | 1259.9 |
| Module2 | MCRS1 | 993.11 |
| Module2 | APP | 2489.3 |
| Module2 | PXN | 1426 |
| Module2 | RPL7 | 649.69 |
| Module2 | CALR | 1067.8 |
| Module2 | ELN | 1318.8 |
| Module2 | FBN1 | 553.73 |
| Module2 | ITGB2 | 243.85 |
| Module2 | NFKBIB | 1929.5 |
| Module2 | TFCP2 | 272.43 |
| Module2 | SAP18 | 424 |
| Module2 | GEMIN4 | 1899.1 |
| Module2 | SUFU | 771.86 |
| Module2 | APBB1 | 830.98 |
| Module2 | BGN | 757.51 |
| Module2 | COL1A1 | 1281 |
| Module2 | DCN | 268.56 |
| Module2 | FBLN1 | 458.15 |
| Module2 | FBLN2 | 323.51 |
| Module2 | FBN2 | 472.76 |
| Module2 | ITGA5 | 800.63 |
| Module2 | LGALS3 | 1604.8 |
| Module2 | LGALS3BP | 632.49 |
| Module2 | LTBP1 | 789.7 |
| Module2 | MDK | 255.67 |
| Module2 | PRTN3 | 284.46 |
| Module2 | SGCA | 305.98 |
| Module2 | AIFM1 | 1204.9 |
| Module2 | NID2 | 253.17 |
| Module2 | AKTIP | 324.73 |
| Module2 | NOL12 | 213 |
| Module2 | COL18A1 | 512.43 |
| Module2 | ASS1 | 573.43 |
| Module2 | LYZ | 318.24 |
| Module2 | PTP4A3 | 916.77 |
| Module2 | POLR1D | 424 |
| Module3 | F3 | 98.02 |
| Module3 | KPNB1 | 851.86 |
| Module3 | EPOR | 993.06 |
| Module3 | HSPA8 | 3846.8 |
| Module3 | RGS2 | 631.46 |
| Module3 | ACVR1 | 1018 |
| Module3 | ANXA7 | 0 |
| Module3 | ATP5B | 727.5 |
| Module3 | CLTA | 0 |
| Module3 | FNTA | 137.66 |
| Module3 | HLA-G | 456.39 |
| Module3 | KIR2DL4 | 0 |
| Module3 | PSMD13 | 1215 |
| Module3 | TAF15 | 91.338 |
| Module3 | SFRS11 | 0 |
| Module3 | TRAP1 | 171.16 |
| Module3 | ATPAF1 | 0 |
| Module3 | VPS29 | 0 |
| Module3 | CMYA5 | 0 |
| Module3 | ZNF512B | 175.22 |
| Module3 | EPO | 0 |
| Module3 | MTHFD2 | 0 |
| Module3 | IGF1R | 1020.1 |
| Module3 | SMAD2 | 4977.9 |
| Module3 | SMAD4 | 3154.8 |
| Module3 | SRI | 212 |
| Module3 | YWHAG | 2009 |
| Module3 | RPS27A | 1918.7 |
| Module3 | TGFB1 | 1440.4 |
| Module3 | CBL | 1585.7 |
| Module3 | CRK | 817.54 |
| Module3 | STUB1 | 863.06 |
| Module3 | COPB1 | 369.73 |
| Module3 | IGF1 | 494.01 |
| Module3 | IGSF1 | 727.78 |
| Module3 | INHBA | 549.01 |
| Module3 | SMAD5 | 1337.5 |
| Module3 | SMAD7 | 889.4 |
| Module3 | PDGFRB | 1539 |
| Module3 | PSMC4 | 555.66 |
| Module3 | UBE2D1 | 212 |
| Module3 | UBE2L3 | 621.92 |
| Module3 | YWHAE | 1054.3 |
| Module3 | TOB1 | 703.03 |
| Module3 | FNBP1 | 110.97 |
| Module3 | PIAS4 | 530.01 |
| Module3 | ACVR1B | 1247.8 |
| Module3 | ACVR2B | 689.53 |
| Module3 | CISH | 462.75 |
| Module3 | ENG | 654.58 |
| Module3 | IGFBP7 | 477.91 |
| Module3 | INHBB | 325.41 |
| Module3 | LRP1 | 860.09 |
| Module3 | SNX2 | 617.94 |
| Module3 | TFE3 | 409.13 |
| Module3 | TGFBR2 | 596.55 |
| Module3 | HECTD1 | 187.8 |
| Module3 | SYNJ2BP | 204.85 |
| Module3 | TGFBRAP1 | 367.41 |
| Module3 | PSMD8 | 411.91 |
| Module3 | FBXO3 | 312.93 |
| Module4 | PRMT1 | 1136.6 |
| Module4 | HSPB1 | 2464.1 |
| Module4 | MAPKAPK3 | 703.53 |
| Module4 | EIF4G1 | 461.7 |
| Module4 | MAPK14 | 758.39 |
| Module4 | EIF4EBP1 | 603.52 |
| Module4 | MAPK9 | 770.76 |
| Module4 | PSMD11 | 1637.4 |
| Module4 | PRKD1 | 330.54 |
| Module4 | MAPKAPK5 | 788.93 |
| Module4 | RPS6KA4 | 225.6 |
| Module4 | MED31 | 1018.4 |
| Module4 | AKR1B1 | 2632.2 |
| Module4 | MYH11 | 0 |
| Module5 | RAB5A | 731.06 |
| Module5 | RBX1 | 777.05 |
| Module5 | RNF2 | 0 |
| Module5 | CCL7 | 0 |
| Module5 | HBXIP | 0 |
| Module5 | NCOA6 | 691.74 |
| Module5 | TNFAIP8 | 0 |
| Module5 | BRF2 | 554.98 |
| Module5 | ALS2CL | 0 |
| Module5 | PTS | 356.9 |
| Module5 | MED4 | 213 |
| Module5 | FNDC5 | 0 |
| Module5 | OTUD4 | 0 |
| Module5 | FBXO22 | 0 |
| Module5 | ABL1 | 2002.3 |
| Module5 | E2F1 | 627.97 |
| Module5 | TOP1 | 557.5 |
| Module5 | TP53 | 4715.8 |
| Module5 | TSC2 | 1514.6 |
| Module5 | CCNH | 758.54 |
| Module5 | RING1 | 415.09 |
| Module5 | TAF1 | 354.8 |
| Module5 | MAGED1 | 723.54 |
| Module5 | MED9 | 645.12 |
| Module5 | CDKN2A | 585.39 |
| Module5 | NCOA1 | 1456.8 |
| Module5 | SIN3A | 467.76 |
| Module5 | AES | 378.24 |
| Module5 | BCR | 939.09 |
| Module5 | HSPA1A | 993.81 |
| Module5 | MCM7 | 2286.1 |
| Module5 | NEDD8 | 746.94 |
| Module5 | SAT1 | 517.97 |
| Module5 | VDR | 369.54 |
| Module5 | MTA1 | 781.82 |
| Module5 | COPS4 | 409.59 |
| Module5 | AREG | 1165.1 |
| Module5 | HSPA9 | 986.65 |
| Module5 | NTHL1 | 215 |
| Module5 | TEAD1 | 110.01 |
| Module5 | UBE3A | 274.65 |
| Module5 | MBIP | 399.09 |
| Module5 | STX5 | 371.52 |
| Module5 | CHRD | 176.38 |
| Module5 | MMAB | 866.12 |
| Module5 | MYH7 | 95.287 |
| Module6 | ZFYVE9 | 687.73 |
| Module6 | COPS5 | 960.4 |
| Module6 | RPS27L | 140.09 |
| Module6 | BCL6 | 515.68 |
| Module6 | FHL2 | 1655 |
| Module6 | PPARG | 573 |
| Module6 | IQGAP1 | 423.74 |
| Module6 | TFAP2A | 254.23 |
| Module6 | CREBBP | 4052 |
| Module6 | PPP2R1A | 722.01 |
| Module6 | STAT5B | 890.55 |
| Module6 | TBP | 1020.2 |
| Module6 | MPG | 848.29 |
| Module6 | ZBTB16 | 565.91 |
| Module6 | TGS1 | 268.13 |
| Module6 | PSMC2 | 623.7 |
| Module6 | XRCC6 | 1300.3 |
| Module6 | CLTC | 703.32 |
| Module6 | MCM2 | 296.55 |
| Module6 | ATF1 | 407.05 |
| Module6 | ATP6V1E1 | 483.57 |
| Module6 | CDC25B | 654.31 |
| Module6 | EIF2B1 | 179.58 |
| Module6 | MAP3K3 | 1800.5 |
| Module6 | UXT | 418.51 |
| Module6 | CITED2 | 286.86 |
| Module6 | ETS2 | 408.58 |
| Module7 | ACTN4 | 692.92 |
| Module7 | FLNA | 3077.5 |
| Module7 | TM4SF1 | 213.23 |
| Module7 | MDFI | 309.67 |
| Module7 | PRKDC | 1791.2 |
| Module7 | TRIM3 | 0 |
| Module7 | WDR42A | 0 |
| Module7 | ANPEP | 0 |
| Module7 | CAD | 664.79 |
| Module7 | CDC42 | 972.55 |
| Module7 | RRAD | 670.49 |
| Module7 | LMNA | 407.67 |
| Module7 | CAMK2G | 741.2 |
| Module7 | PIK3R1 | 2358.3 |
| Module7 | FRAP1 | 876.34 |
| Module7 | MAP2K4 | 218.77 |
| Module7 | TRAF2 | 1217.3 |
| Module7 | DNAJA1 | 483.31 |
| Module7 | ILF3 | 644.94 |
| Module7 | PDE6D | 201 |
| Module7 | RALA | 255.59 |
| Module7 | TLN1 | 712.64 |
| Module7 | TNFRSF1B | 1070 |
| Module7 | CKAP5 | 1029.7 |
| Module7 | RIPK3 | 1160.6 |
| Module7 | NGFRAP1 | 197.4 |
| Module7 | TNIP2 | 210.04 |
| Module7 | PRR5 | 261.75 |
| Module8 | PTK2 | 1499.7 |
| Module8 | ESR1 | 2587.8 |
| Module8 | TNNI3K | 1643.8 |
| Module8 | ARL2 | 7.8701 |
| Module8 | CALM1 | 2887.6 |
| Module8 | COIL | 822.95 |
| Module8 | BZRAP1 | 0 |
| Module8 | GABARAPL2 | 808.84 |
| Module8 | AURKAIP1 | 780 |
| Module8 | CENPT | 780 |
| Module8 | TSPO | 198 |
| Module8 | DBI | 394 |
| Module8 | NRGN | 0 |
| Module8 | PPCDC | 588 |
| Module8 | ANKS1B | 0 |
| Module8 | UBE2I | 1834.4 |
| Module8 | SQSTM1 | 1378.6 |
| Module8 | CAMK2D | 476.39 |
| Module8 | RIPK2 | 1436.7 |
| Module8 | ACTN1 | 1055.4 |
| Module8 | ACTN2 | 372.53 |
| Module8 | PSMC5 | 948.21 |
| Module8 | EPB41 | 348.25 |
| Module8 | MYH9 | 1294 |
| Module8 | NCL | 238.62 |
| Module8 | S100A4 | 501.61 |
| Module8 | TBCD | 204.02 |
| Module8 | TNNI3 | 760.01 |
| Module8 | TTN | 2248 |
| Module8 | CXCR4 | 574.96 |
| Module8 | UNC119 | 218.53 |
| Module8 | TRAF4 | 411.85 |
| Module8 | SORBS1 | 714.91 |
| Module8 | ACTA1 | 1979.1 |
| Module8 | ANK1 | 300.09 |
| Module8 | CCR5 | 312.37 |
| Module8 | CD163 | 51.095 |
| Module8 | TRIM63 | 329.43 |
| Module8 | MYBPC3 | 624.54 |
| Module8 | PIK3C3 | 588 |
| Module9 | MCL1 | 464.18 |
| Module9 | SCP2 | 200 |
| Module9 | DAD1 | 0 |
| Module9 | ACAA2 | 0 |
| Module9 | PPP2CA | 1214.8 |
| Module9 | MAPK1 | 5408.1 |
| Module9 | APOA1 | 986 |
| Module9 | RHOB | 200 |
| Module9 | CTSL1 | 1667.4 |
| Module9 | DUSP3 | 245.93 |
| Module9 | EHHADH | 398 |
| Module9 | IGFBP3 | 455.72 |
| Module9 | GATA4 | 1009.3 |
| Module9 | HAND2 | 519.12 |
| Module9 | IFNAR1 | 569.44 |
| Module9 | PPP1CA | 1210.6 |
| Module9 | FOS | 642.95 |
| Module9 | TBX5 | 113.4 |
| Module10 | GRB2 | 5613.6 |
| Module10 | NFKBIA | 1641.2 |
| Module10 | PAK1 | 1465.5 |
| Module10 | RAF1 | 2132.8 |
| Module10 | TNFRSF1A | 1341.4 |
| Module10 | WIPF1 | 359.39 |
| Module10 | DYNLL1 | 3223.2 |
| Module10 | SPRY4 | 0 |
| Module10 | ACTB | 2448.5 |
| Module10 | ACTG1 | 1640.3 |
| Module10 | AP2A1 | 373.56 |
| Module10 | BMP4 | 207.81 |
| Module10 | CSF1R | 423.12 |
| Module10 | DAG1 | 819.73 |
| Module10 | DMD | 272.53 |
| Module10 | FYN | 1527.8 |
| Module10 | MCC | 1132.9 |
| Module10 | MIF | 947.81 |
| Module10 | PAX6 | 196 |
| Module10 | PLCG1 | 1881.4 |
| Module10 | PPP2R2A | 410.63 |
| Module10 | PRKACA | 1918.9 |
| Module10 | IRS4 | 421.5 |
| Module10 | PDCD6 | 196 |
| Module10 | NUDT21 | 978.13 |
| Module10 | CCT5 | 269.98 |
| Module10 | CYCS | 1473.5 |
| Module10 | PPP1R9B | 317.05 |
| Module10 | ACTC1 | 2844.2 |
| Module10 | CIRBP | 333.54 |
| Module10 | CYBB | 0 |
| Module10 | DDX3X | 720.21 |
| Module10 | CCR10 | 346.99 |
| Module10 | HMGN1 | 0 |
| Module10 | INPPL1 | 119.68 |
| Module10 | MYOC | 1153.4 |
| Module10 | RPS6KB1 | 329.32 |
| Module10 | FSCN1 | 1237.1 |
| Module10 | SOD2 | 0 |
| Module10 | AURKA | 853.17 |
| Module10 | STX4 | 727.85 |
| Module10 | TRIP6 | 804.17 |
| Module10 | UBE2D2 | 196 |
| Module10 | VCL | 3189.1 |
| Module10 | VDAC1 | 1633.2 |
| Module10 | CNTNAP1 | 21.013 |
| Module10 | SNAP29 | 0 |
| Module10 | STAMBP | 196 |
| Module10 | CLPX | 196 |
| Module10 | WWP1 | 409.44 |
| Module10 | SYNE1 | 428.57 |
| Module10 | UNC13D | 332.76 |
| Module10 | PPP2CB | 1137.9 |
| Module10 | HTRA1 | 327.03 |
| Module10 | VPS72 | 622.07 |
| Module10 | C20orf24 | 499.26 |
| Module10 | DYNC1H1 | 1243.9 |
| Module10 | EIF1B | 1309.7 |
| Module10 | BRP44L | 0 |
| Module10 | CHD6 | 224.36 |
| Module10 | CYC1 | 196 |
| Module10 | ACAT1 | 0 |
| Module10 | RAB21 | 0 |
| Module10 | REXO2 | 113.81 |
| Module11 | CSNK2B | 1334.5 |
| Module11 | SRC | 1983.6 |
| Module11 | RAB1A | 592.48 |
| Module11 | SREBF2 | 634.14 |
| Module11 | CRH | 0 |
| Module11 | LEP | 609 |
| Module11 | UCN | 408 |
| Module11 | INSIG2 | 0 |
| Module11 | VTA1 | 330.8 |
| Module11 | ZNF44 | 0 |
| Module11 | SOX10 | 84.889 |
| Module11 | WAS | 1305.6 |
| Module11 | CRHR2 | 205 |
| Module11 | STAT3 | 1656.1 |
| Module11 | HSP90AA1 | 2423.5 |
| Module11 | XRCC1 | 640.52 |
| Module11 | BRF1 | 298.91 |
| Module11 | TCF3 | 1089.1 |
| Module11 | RAC1 | 798.47 |
| Module11 | SUMO1 | 745.92 |
| Module11 | CSNK2A1 | 5778 |
| Module11 | SRF | 689.69 |
| Module11 | CDKN1A | 1535.2 |
| Module11 | RANGAP1 | 583.08 |
| Module11 | CDC37 | 968.31 |
| Module11 | CD8A | 237.55 |
| Module11 | NKX2-5 | 163.03 |
| Module11 | DVL3 | 685.5 |
| Module11 | ERH | 1230 |
| Module11 | PSMA3 | 906.85 |
| Module11 | SNCA | 471.45 |
| Module11 | VTN | 408 |
| Module11 | HIPK2 | 517.33 |
| Module11 | CAMK2A | 523.94 |
| Module11 | CASP7 | 192.29 |
| Module11 | EIF5 | 400.75 |
| Module11 | NOLC1 | 338.4 |
| Module11 | KLHL12 | 626.48 |
| Module11 | SFRS12 | 275.17 |
| Module11 | AHSA1 | 609 |
| Module11 | DIDO1 | 306.13 |
| Module12 | B2M | 1983.3 |
| Module12 | AP2M1 | 400 |
| Module12 | RAN | 892.77 |
| Module12 | RELA | 4456.2 |
| Module12 | TRAF6 | 4047.1 |
| Module12 | EPHB6 | 121.52 |
| Module12 | EWSR1 | 1808.2 |
| Module12 | TSC22D1 | 1644.1 |
| Module12 | DTNBP1 | 1052.1 |
| Module12 | A2M | 1602.2 |
| Module12 | CD74 | 201 |
| Module12 | EIF1AX | 253.54 |
| Module12 | MRPL12 | 0 |
| Module12 | MAPK8IP3 | 512.55 |
| Module12 | VPS24 | 541.38 |
| Module12 | NDUFV1 | 0 |
| Module12 | GSK3B | 2974.8 |
| Module12 | SMAD1 | 1589.5 |
| Module12 | BAT3 | 2538.2 |
| Module12 | UBB | 1316.9 |
| Module12 | PDHX | 204.12 |
| Module12 | IKBKG | 3009.4 |
| Module12 | BMPR1A | 205.2 |
| Module12 | STX1A | 560.59 |
| Module12 | PHB2 | 1141.2 |
| Module12 | CD44 | 1063.4 |
| Module12 | MYOD1 | 745.76 |
| Module12 | PSEN1 | 1272.6 |
| Module12 | RAB11A | 1639.3 |
| Module12 | BLOC1S1 | 641.92 |
| Module12 | NFKB1 | 969.89 |
| Module12 | APC | 550.64 |
| Module12 | NOTCH1 | 3010.6 |
| Module12 | SNW1 | 869.99 |
| Module12 | FURIN | 597 |
| Module12 | NUMB | 434.1 |
| Module12 | LNX1 | 539.61 |
| Module12 | MATR3 | 344.59 |
| Module12 | ARL4D | 399.67 |
| Module12 | TJP1 | 467.64 |
| Module12 | JAG1 | 841.65 |
| Module12 | MLLT4 | 731.13 |
| Module12 | MMP9 | 718.74 |
| Module12 | NOTCH2 | 1189.3 |
| Module12 | NOTCH3 | 563.34 |
| Module12 | THBS1 | 1014.6 |
