# Supplementary material for: Identification of dysfunctional modules and disease genes in congenital heart disease by a network-based approach
Source: BMC Genomics. 2011 Dec 2;12:592. doi: 10.1186/1471-2164-12-592 (PMC3256240; doi:10.1186/1471-2164-12-592)
Supplement: Additional file 3 — Pathways enriched in CHD subnetwork using hypergeometric test. Top 12 pathways are used for classification evaluation. [file 1471-2164-12-592-S3.DOC]

### Additional File 3: Pathways enriched in CHD subnetwork using hypergeometric test. Top 12 pathways are used for classification evaluation.

**Table A3.1. Pathways enriched in CHD subnetwork using hypergeometric test. Top 12 pathways are used for classification evaluation.**

| **KEGG ID** | **P value** | **Odds Ratio** | **ExpCount** | **Count** | **Size** | **Term** |
| --- | --- | --- | --- | --- | --- | --- |
| 4012 | 2.63E-14 | 10.71 | 6.61 | 29 | 47 | ErbB signaling pathway |
| 4722 | 6.19E-14 | 6.76 | 10.40 | 37 | 74 | Neurotrophin signaling pathway |
| 4520 | 2.68E-13 | 9.76 | 6.61 | 28 | 47 | Adherens junction |
| 4350 | 2.50E-12 | 8.90 | 6.61 | 27 | 47 | TGF-beta signaling pathway |
| 4330 | 2.23E-07 | 9.89 | 3.23 | 14 | 23 | Notch signaling pathway |
| 4660 | 6.73E-07 | 4.37 | 8.01 | 23 | 57 | T cell receptor signaling pathway |
| 4510 | 8.58E-07 | 3.01 | 16.16 | 36 | 115 | Focal adhesion |
| 4912 | 2.04E-06 | 4.59 | 6.75 | 20 | 48 | GnRH signaling pathway |
| 4010 | 3.51E-06 | 2.62 | 19.82 | 40 | 141 | MAPK signaling pathway |
| 4310 | 4.51E-06 | 3.30 | 11.24 | 27 | 80 | Wnt signaling pathway |
| 4720 | 7.10E-06 | 5.61 | 4.50 | 15 | 32 | Long-term potentiation |
| 4114 | 1.60E-05 | 3.64 | 8.15 | 21 | 58 | Oocyte meiosis |
| 4916 | 3.82E-05 | 3.82 | 6.75 | 18 | 48 | Melanogenesis |
| 4910 | 5.49E-05 | 2.97 | 10.68 | 24 | 76 | Insulin signaling pathway |
| 4370 | 5.91E-05 | 4.06 | 5.76 | 16 | 41 | VEGF signaling pathway |
| 4730 | 7.40E-05 | 5.40 | 3.65 | 12 | 26 | Long-term depression |
| 4670 | 7.67E-05 | 3.27 | 8.29 | 20 | 59 | Leukocyte transendothelial migration |
| 4110 | 9.64E-05 | 3.00 | 9.70 | 22 | 69 | Cell cycle |
| 4530 | 9.64E-05 | 3.00 | 9.70 | 22 | 69 | Tight junction |
| 4662 | 1.23E-04 | 3.96 | 5.48 | 15 | 39 | B cell receptor signaling pathway |
| 4320 | 1.95E-04 | 8.33 | 1.97 | 8 | 14 | Dorso-ventral axis formation |
| 4740 | 1.95E-04 | 8.33 | 1.97 | 8 | 14 | Olfactory transduction |
| 4920 | 2.55E-04 | 3.84 | 5.20 | 14 | 37 | Adipocytokine signaling pathway |
| 4062 | 2.61E-04 | 2.49 | 13.07 | 26 | 93 | Chemokine signaling pathway |
| 4621 | 2.66E-04 | 4.10 | 4.64 | 13 | 33 | NOD-like receptor signaling pathway |
| 4664 | 7.19E-04 | 3.56 | 5.06 | 13 | 36 | Fc epsilon RI signaling pathway |
| 4612 | 1.13E-03 | 3.15 | 5.90 | 14 | 42 | Antigen processing and presentation |
| 4210 | 1.88E-03 | 2.94 | 6.18 | 14 | 44 | Apoptosis |
| 4144 | 2.00E-03 | 2.03 | 16.30 | 28 | 116 | Endocytosis |
| 4622 | 2.18E-03 | 3.45 | 4.36 | 11 | 31 | RIG-I-like receptor signaling pathway |
| 4914 | 2.20E-03 | 3.02 | 5.62 | 13 | 40 | Progesterone-mediated oocyte maturation |
| 4810 | 3.43E-03 | 1.99 | 15.32 | 26 | 109 | Regulation of actin cytoskeleton |
| 4650 | 3.88E-03 | 2.46 | 8.01 | 16 | 57 | Natural killer cell mediated cytotoxicity |
| 4620 | 4.70E-03 | 2.48 | 7.45 | 15 | 53 | Toll-like receptor signaling pathway |
| 4666 | 5.59E-03 | 2.63 | 6.18 | 13 | 44 | Fc gamma R-mediated phagocytosis |
| 4270 | 2.04E-02 | 2.14 | 7.17 | 13 | 51 | Vascular smooth muscle contraction |
| 4115 | 2.25E-02 | 2.39 | 5.06 | 10 | 36 | p53 signaling pathway |
| 4962 | 2.76E-02 | 2.43 | 4.50 | 9 | 32 | Vasopressin-regulated water reabsorption |
| 4630 | 3.33E-02 | 1.82 | 9.98 | 16 | 71 | Jak-STAT signaling pathway |
| 4150 | 3.38E-02 | 2.48 | 3.94 | 8 | 28 | mTOR signaling pathway |
| 3320 | 4.14E-02 | 2.55 | 3.37 | 7 | 24 | PPAR signaling pathway |
| 4623 | 4.14E-02 | 2.55 | 3.37 | 7 | 24 | Cytosolic DNA-sensing pathway |
