# Supplementary figures and images for: Identification of dysfunctional modules and disease genes in congenital heart disease by a network-based approach
Source: BMC Genomics. 2011 Dec 2;12:592. doi: 10.1186/1471-2164-12-592 (PMC3256240; doi:10.1186/1471-2164-12-592)

Module 4

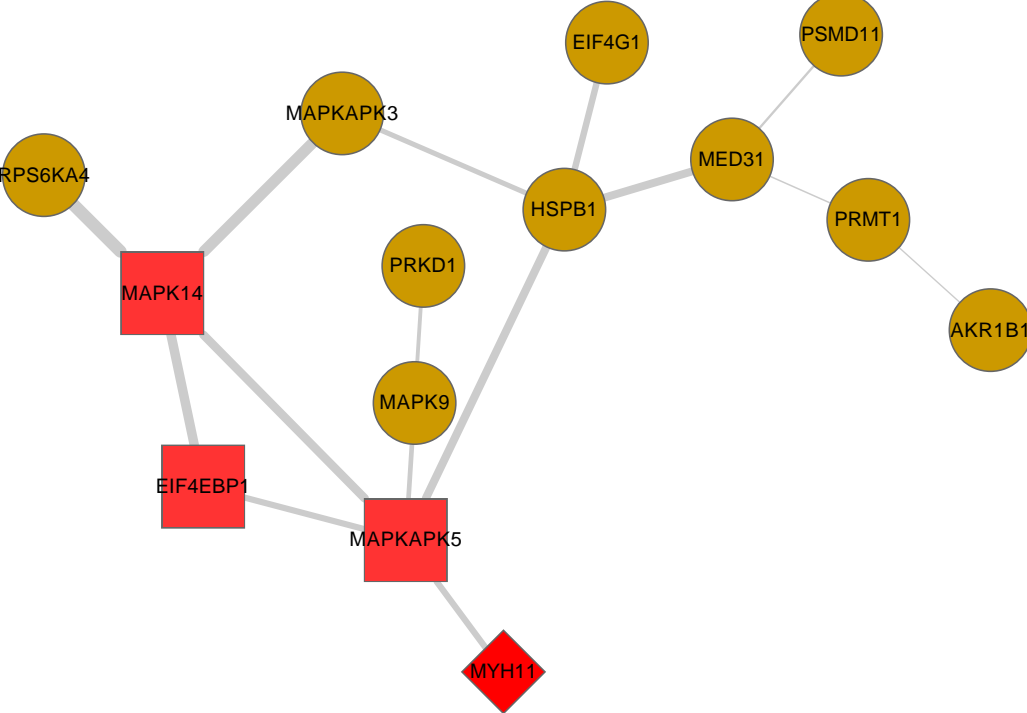

Module 9

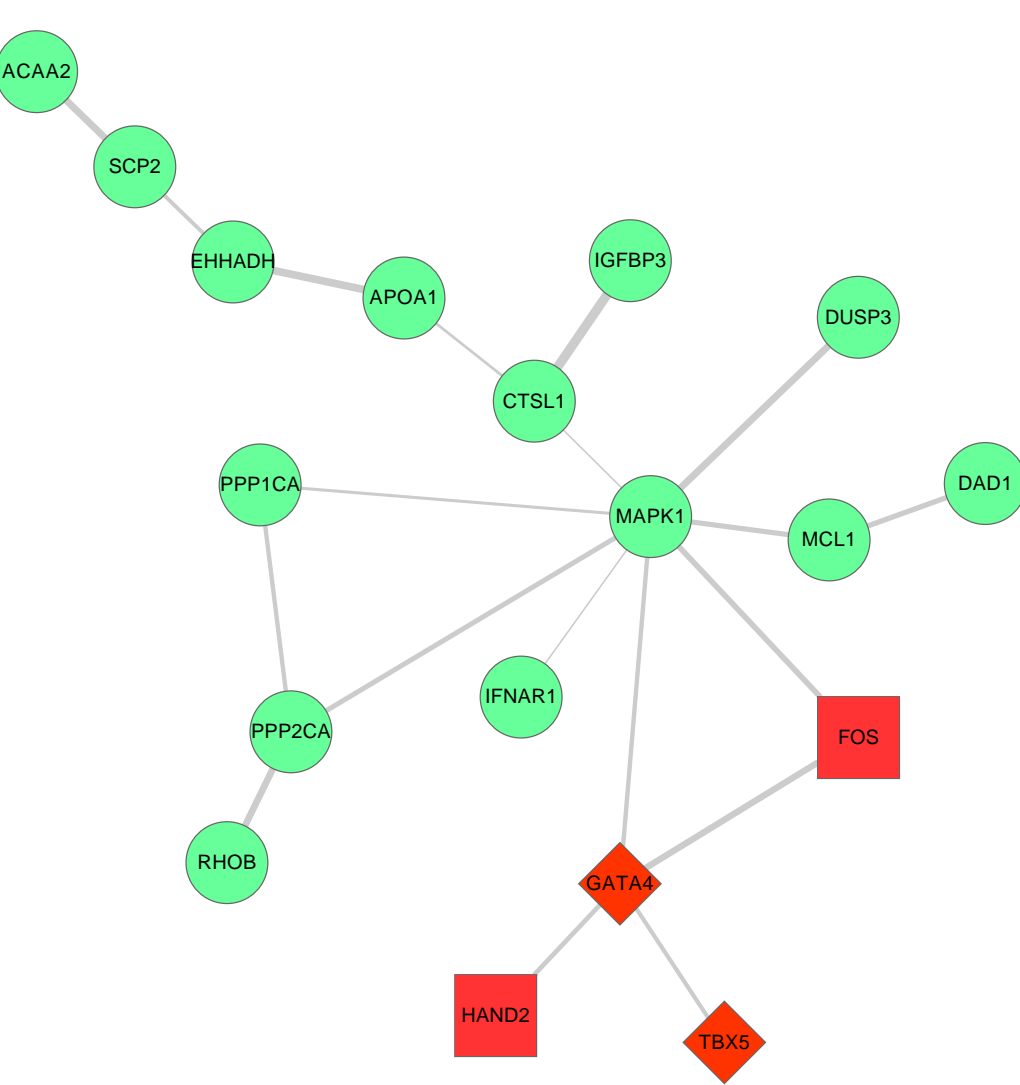

Module 5

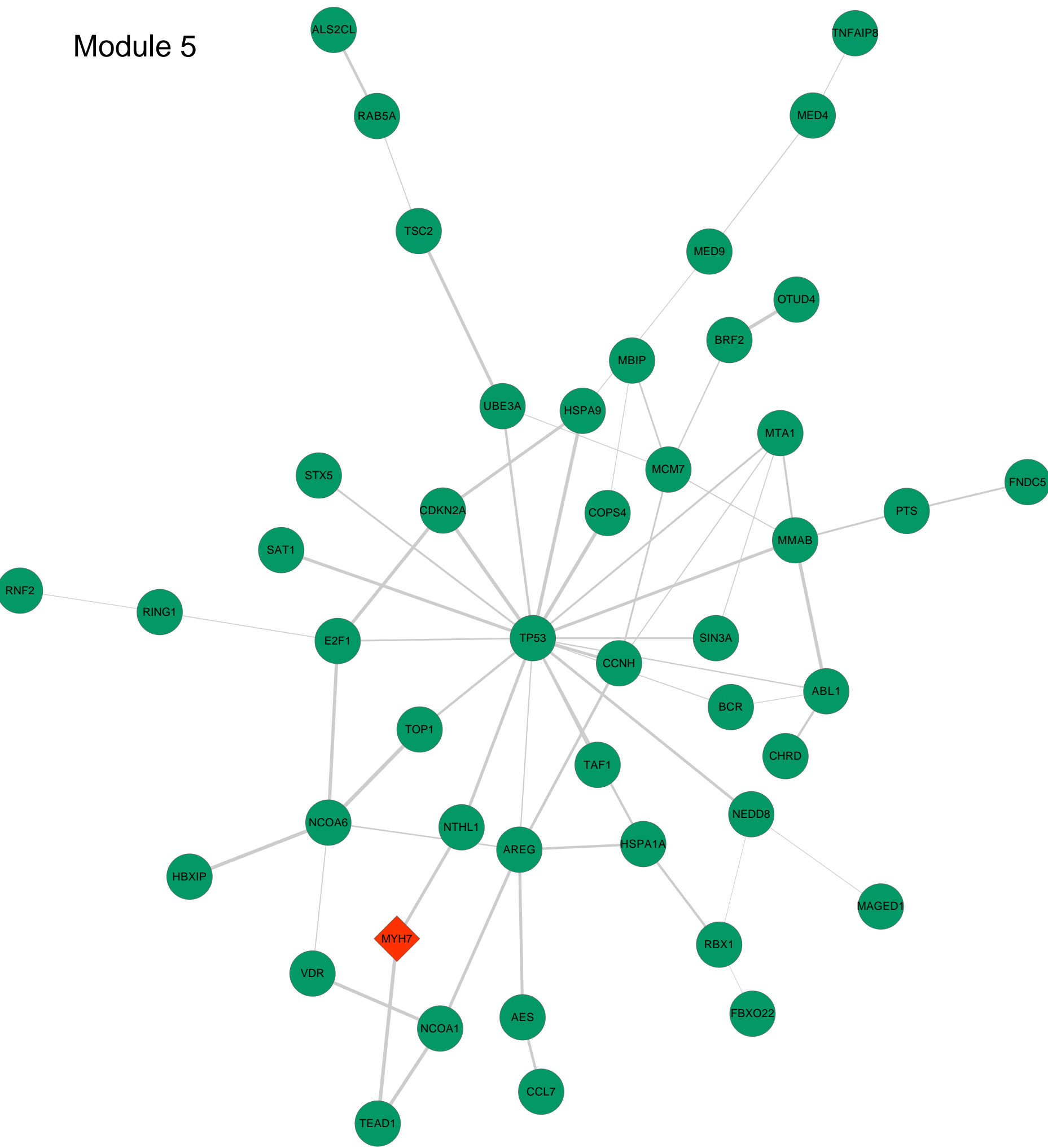

Supplement: Additional file 5 — Network view of Modules 4, 5 and 9. [file 1471-2164-12-592-S5.PDF]
