# Supplementary material for: Identification of dysfunctional modules and disease genes in congenital heart disease by a network-based approach
Source: BMC Genomics. 2011 Dec 2;12:592. doi: 10.1186/1471-2164-12-592 (PMC3256240; doi:10.1186/1471-2164-12-592)
Supplement: Additional file 6 — Details of prioritizing of candidate disease genes by module analysis and robustness analysis of identified modules. [file 1471-2164-12-592-S6.DOC]

### Additional File 6: Supplementary text

### Text 1: Details of prioritizing of candidate disease genes by module analysis

There are 14 known CHD genes which have been tested on the gene expression profile and can be mapped to the PPI network, and all of them are included in the CHD subnetwork. We defined candidate disease genes as those that are either on the shortest paths connecting 14 causal disease genes or the first interacting neighbors of them in the CHD subnetwork. Together there are 114 such genes. Then we assigned a score to each of these candidates, with investigation of its module membership and its GO semantic similarity with the causal disease genes in that particular module. Since a module is considered as a basic functional unit, we reasoned that if a gene interacts with disease genes or on their shortest paths, and have similar GO terms (excluding evidence code IEA - Inferred from Electronic Annotation) with disease genes in the same module, they are more likely to participate in the same biological processes as disease genes. This step results in a primary list of 114 candidate disease genes.

Besides close relationship with known causal disease genes in terms of network distance and functional similarity, the network position of the gene itself needs be taken into consideration. There are already many studies focusing on the network properties of disease genes, and various statistical or topological features have been used, among which node degree and edge betweenness are two fundamental metrics . However, degree measures the number of direct binding partners of a protein and does not consider its position relative to others. Betweenness measures the total number of shortest paths that go through a protein, but does not consider all alternative paths of protein interactions. Also, both of them are pure topological features without considering the confidence scores of protein interactions in specific conditions.

To overcome the above shortcomings, we adopted the concept of current flow from previous study to measure each protein on the interaction network. To assess the overall importance of a gene, we considered both the current flow of a gene in each module and the number of dysfunctional modules that it participates in. Therefore, we summed up the current flow in each of its participating module as an information score to measure its importance. We compared the score of CHD disease genes with that of 500 random gene sets of the same size, and found that CHD genes have significantly lower (Mann-Whitney test, *p*-value=0.0018) score than random average (Figure A6.1), which means they tend to be in less important positions in a module and participate in fewer number of modules. Results of previous studies show that genes harboring human inherited disease mutations are not hub genes and are less likely to play essential roles compared with genes on global network. We therefore reasoned whether similar explanations could be applied to our findings as well, and whether mutations in highly scored genes can be lethal for embryos, because such genes tend to be in central position of a module or have influence to many modules, allowing large amount of information flow to pass through it. If the embryo even cannot develop to term as a result, such mutations will not be observed in human population. In contrast, if a gene locates in marginal space with less important role in information passage, its negative influence is much smaller, and the embryos might survive to birth, then the disease phenotype can be observed. With this in mind, we required the information score of a candidate gene not significantly different (*p*-value<0.05) from that of CHD genes, and this results in a final list of 60 candidate disease genes.

To measure the functional similarity between two genes in terms of GO annotations, we adopted the method developed by Wang et al. , which encodes a GO term’s semantics into a numeric value by aggregating the semantic contributions of their ancestor terms (including this specific term) in the GO directed graph. We extracted all GO terms associated with two genes (evidence code IEA is excluded), and compared the similarity of the two sets of GO terms. The similarity between a GO term$go$ and a gene$G1$ is defined as the similarity between the GO term $go$ and all the GO terms associated with the gene$G1$,

where are the GO terms associated with the gene$G1$.

The similarity between two genes $G1$ and $G2$is defined as

where and are the GO terms associated with the genes $G1$and$G2$, respectively.

**Text 2: Robustness of module identification**

Our identified modules are based on the 14 source genes. To analyze the robustness of our module identification result when only use a subset of source genes, we randomly sample 10 source genes and identify modules using the same procedure and parameter setting as described in the main text. Then we computed the overlap of this testing set (modules identified from subset of source genes) with the reference set (modules identified from full set of source genes) in a pair-wise manner by hypergeometric test, and for each reference module we choose the minimum p-value. Figure A6.2 is an example of the significance of overlap between a randomly selected set of testing modules (y-axis, “test_”) and the reference set of modules (x-axis, “ref_”), each module is labeled with one source gene symbol as its name. We generated 100 alternative sets of testing modules and computed the overlap with the reference modules. As is shown in Figure A6.3, except Module6, all other modules have consistent significant overlap with testing modules (p-value<1E-10). This indicates that, although eliminating source genes results in fewer modules, the composition of each module is, in general, independent of the subset of source genes used. Such robust performance has to do with our heuristic approach in computing information score—we identify the shortest paths from a source gene to all target genes, and compute information score in this subnetwork, which means the information score for each gene in this subnetwork is only related to a particular source gene. The choice of subsets of source genes changes available subnetworks, and can influence the result during the module identification procedure, in which a gene is reassigned to the modules which maximize its information score while maintaining connectivity.

**Figure A6.1. Network features of CHD genes and random gene sets.**

**Figure A6.2. An example of the significance of overlap between reference set of modules (x-axis) and a random testing set of modules (y-axis). 12 reference modules are identified from 14 source genes while 10 modules are identified from 10 source genes, where 7 of them have significant overlap (p-value<0.001) with reference modules.**

**Figure A6.3. Significance of overlap between reference modules and 100 alternative sets of testing modules generated from subsets of source genes. Except M6 (which may be due to its size), all other modules show significant recapitulation (p-value<1E-10) under 100 random iterations.**

**References:**

1. Barabási AL, Oltvai ZN: Network biology understanding the cell's functional organization. *Nature Reviews Genetics* 2004, 5(2):101-113.

2. Missiuro PV, Liu K, Zou L, Ross BC, Zhao G, Liu JS, Ge H: Information Flow Analysis of Interactome Networks. *PLoS Comput Biol* 2009, 5(4):e1000350.

3. Feldman I, Rzhetsky A, Vitkup D: Network properties of genes harboring inherited disease mutations. *Proc Natl Acad Sci U S A* 2008, 105(11):4323-4328.

4. Goh KI, Cusick ME, Valle D, Childs B, Vidal M, Barabási AL: The human disease network. *Proc Natl Acad Sci U S A* 2007, 104:8685-8690.

5. Wang JZ, Du Z, Payattakool R, Yu PS, Chen CF: A new method to measure the semantic similarity of GO terms. *Bioinformatics* 2007, 23(10):1274-1281.

**Table A6.1. Full list of final candidate disease genes.**

| **Gene** | **Score** |
| --- | --- |
| HAND2 | 6.87 |
| FOS | 4.09 |
| NOTCH2 | 2.5 |
| MLLT4 | 1.72 |
| THBS1 | 1.7 |
| MAPK14 | 1.69 |
| ELK1 | 1.56 |
| MAPKAPK5 | 1.54 |
| DCN | 1.52 |
| NUMB | 1.49 |
| NFKB1 | 1.45 |
| RALA | 1.43 |
| EIF4EBP1 | 1.25 |
| TRAF2 | 1.25 |
| FURIN | 1.22 |
| BGN | 1.16 |
| SNW1 | 1.15 |
| TGFBRAP1 | 1.13 |
| FBN2 | 1.11 |
| ACVR1B | 1.08 |
| TLN1 | 1.05 |
| BLOC1S1 | 1.01 |
| RIPK3 | 1.01 |
| TJP1 | 0.98 |
| TFAP2A | 0.95 |
| HIPK2 | 0.89 |
| IGSF1 | 0.84 |
| SGCA | 0.8 |
| FSCN1 | 0.79 |
| ENG | 0.78 |
| FBN1 | 0.78 |
| CRK | 0.77 |
| PDGFRB | 0.74 |
| INHBA | 0.74 |
| CAMK2G | 0.73 |
| MYH9 | 0.7 |
| INHBB | 0.68 |
| LMNA | 0.67 |
| PRTN3 | 0.64 |
| SYNE1 | 0.63 |
| UXT | 0.6 |
| WIPF1 | 0.56 |
| LYZ | 0.48 |
| ACTN2 | 0.4 |
| DMD | 0.4 |
| SNX2 | 0.36 |
| PPP1R9B | 0.31 |
| VPS72 | 0.12 |
| PSEN2 | 0 |
| CRIP2 | 0 |
| FBLN1 | 0 |
| LGALS3 | 0 |
| NOTCH3 | 0 |
| PIAS1 | 0 |
| TNFRSF1B | 0 |
| LEF1 | 0 |
| FBLN2 | 0 |
| NID2 | 0 |
| ASS1 | 0 |
| PRR5 | 0 |
